# Xylityl Sesquicaprylate Efficacy as an Antiseptic Ingredient for Oral Care Products (Mouthwash): An In Vitro Screening Investigation against Eight Microorganisms

**DOI:** 10.3390/molecules28010028

**Published:** 2022-12-21

**Authors:** Cecília Nogueira, Liliam Mussi, André Rolim Baby, Roseli Zupeli, Wagner Vidal Magalhães

**Affiliations:** 1 Research and Development Department, Chemyunion Ltd., Sorocaba 18087-101, Brazil; 2 Department of Pharmacy, Faculty of Pharmaceutical Sciences, University of São Paulo, São Paulo 05508-000, Brazil; 3 Research and Development Department, Phisalia Produtos de Beleza Ltd., Osasco 06276-000, Brazil

**Keywords:** alcohol-free mouthwash, time-kill test, triclosan, xylityl sesquicaprylate

## Abstract

For dental caries and periodontal diseases initiated by dental plaque (as bacterial communities) and to inhibit the growth of oral pathogenic bacteria, oral care products containing antiseptic active ingredients are highly recommended, nonetheless, side effects of such actives are a concern (teeth discoloration/staining and taste perception, for example). In this context, we challenged xylityl sesquicaprylate, an antiseptic compound from natural resources, as an active ingredient to be used in an alcohol-free mouthwash formulation. The xylityl sesquicaprylate sample was compared to a respective blank mouthwash formulation and one containing triclosan. The in vitro efficacy was screened by the time-kill assay against eight microorganisms. The xylityl sesquicaprylate-containing mouthwash (0.45% *w*/*w*) presented a particularly interesting profile of efficacy against *Actinomyces viscosus*, *Fusobacterium nucleatum*, *Porphyromonas gingivalis*, and *Tannerella forsythia*, with results of greater magnitude to reduce the log_10_ of those microorganisms in comparison with the triclosan sample.

## 1. Introduction

Widely recognized as an expensive approach, the treatment of dental diseases must be convenient to access, act as a preventive strategy to keep healthy, and be cost-effective, since periodontal disorders are a worldwide problem of public health. In addition to function commitment(s), periodontitis can cause discomfort and aesthetic concerns [1].

Dental caries and periodontal diseases are initiated by dental plaque (as bacterial communities), with *Streptococcus mutans* acting as one of the causative agents. To inhibit the growth of the oral pathogenic bacteria, the oral care products containing antiseptics (chlorhexidine, hydrogen peroxide, and triclosan, among others) are highly recommended; nonetheless, some side effects of such actives are a concern, such as teeth discoloration or staining and taste perception [2,3]. The marketing of triclosan in bacterial soaps, for instance, was banned by the Food and Drug Administration, since there was an increasing concern about its presence in the environment and its impacts on the health of beings [4].

Accordingly, aiming also to elevate the compliance rate of consumers and patients to the regular use of mouthwashes as effective oral care products, there is a real demand in the cosmetic industry to develop safe and effective antiseptics, preferentially from natural and/or renewable resources that will reinforce a robust trend claim for innovative products [5].

In this investigation, we challenged xylityl sesquicaprylate, an antiseptic compound produced from natural resources, as an active ingredient to be used in an alcohol-free mouthwash formulation. The xylityl sesquicaprylate sample was compared to a respective blank mouthwash formulation and one containing triclosan. The in vitro efficacy was screened by the time-kill assay against eight (08) microorganisms.

## 2. Results and Discussion

Table 1 describes our results from the time-kill kinetic test, expressed in log_10_ reductions (*n* = 3), using the eight (08) selected microorganisms against the blank, triclosan 0.03% *w*/*w* and xylityl sesquicaprylate 0.45% *w*/*w* alcohol-free mouthwash samples.

According to Chandashekar and coworkers, 2019, the commonest microorganisms responsible for dental caries/periodontal disorders are *Streptococcus mutans*, *Streptococcus sobrinus*, *Actinobacillus actinomycetemcomitans*, *Fusobatecrium nucleatum,* and *Porphyromonas gingivalis* [1]. The xylityl sesquicaprylate sample performed a particularly high efficacy in the time-kill kinetic test against the *A. viscosus*, *F. nucleatum*, *P. gingivalis,* and *T. forsythia*. For these microorganisms, the xylityl sesquicaprylate sample, at 0.45% *w*/*w*, developed better results in comparison to the blank mouthwash and the one containing triclosan at 0.03% *w*/*w* for both periods of time (30 and 60 s).

When *K. aerogenes* and *P. intermedia* were the tested microorganisms, the xylityl sesquicaprylate-containing mouthwash also developed an effective action, nevertheless, the response was noticed for just one of the periods of time from the time-kill test (60 s for *K. aerogenes*; 30 s for *P. intermedia*). In this specific scenario, the xylityl sesquicaprylate sample presented a narrow efficacy profile against *K. aerogenes*; blank and triclosan samples, as well, generated low log_10_ reductions, ranging from 0.01 to 0.05. The challenged biodegradable antiseptic active, unexpectedly, reduced the log_10_ for *P. intermedia* only for the lowest time of contact, being the triclosan-containing mouthwash more effective in this experimental condition.

The blank formulation developed a better efficacy profile solely against *S. mutans* after 30 s contact, followed by the triclosan and xylityl sesquicaprylate samples, which results were of inferior values. When the contact time was 60 s, the formulation containing triclosan showed the best efficacy. The *C. albicans* was not affected by any of the treatments.

Xylityl sesquicaprylate is a biodegradable antiseptic compound obtained from xylitol and caprylic acid, both of vegetable origin. Xylitol is a penthiol sugar whose main source of industrial production is the use of cereal by-products (corn cob is an example). Caprylic acid is obtained from vegetable oils sustainably extracted and certified by the Roundtable on Sustainable Palm Oil [6]. In addition to being from renewable sources, the synthetic route of xylityl sesquicaprylate was developed aiming to meet the maximum requirements of an ecologically correct process, generating a biodegradable molecule. The xylityl sesquicaprylate’s sugar ester is a non-ionic surfactant consisting of a carbohydrate, as the hydrophilic polar group, and one or more fatty acids, as the lipophilic component(s) of the chemical structure. Having an amphipathic characteristic, xylityl sesquicaprylate can be in the aqueous phase and at the water-oil interface of formulations, ideally acting in the regions of growth of microorganisms. Furthermore, due to its chemical structure, xylityl sesquicaprylate has complementary properties, being able to act as an emollient, solubilizer, and co-emulsifier/surfactant [6,7].

The mouthwash sample added of the xylityl sesquicaprylate at 0.45% *w*/*w* was able to reduce the log_10_ of several microorganisms used herein, such as gram-positive and gram-negative bacteria. Such microorganisms may be found in the oral cavity, being able to adhere over the teeth surface, and they may interact negatively with the global health of the mouth promoting the development and/or aggravation of various periodontal diseases [1]. Unpredictably, considering the *S. mutans* property to cause caries and to reside in biofilms on the teeth surface, both antiseptics (triclosan 0.03% *w*/*w* and xylityl sesquicaprylate 0.45% *w*/*w*) were not adequality effective in reducing the mentioned microorganism log_10_ in the time-kill assay compared to the blank mouthwash. It is noteworthy to mention that triclosan, for that specific microorganism, had a slightly higher performance in regard to the xylityl sesquicaprylate, still inferior to the blank sample.

## 3. Materials and Methods

### 3.1. Samples

Alcohol-free mouthwash samples’ compositions are described in Table 2. Three samples were prepared (blank, triclosan 0.03% *w*/*w* and xylityl sesquicaprylate 0.45% *w*/*w*) and challenged by the time-kill kinetic test. The xylityl sesquicaprylate concentration was established in accordance with a previously minimum inhibitory concentration (MIC) assay performed by [6].

### 3.2. Microorganisms

The following microorganisms were used in our investigation: *Actinomyces viscosus* (ATCC #15987), *Candida albicans* (ATCC #10231), *Fusobacterium nucleatum* (ATCC #25586), *Klebsiella aerogenes* (ATCC #13048), *Porphyromonas gingivalis* (ATCC #33277), *Prevotella intermedia* (ATCC #25611), *Streptococcus mutans* (ATCC #25175), and *Tannerella forsythia* (ATCC #43037).

### 3.3. Time-Kill Test

Based on Mussi and coworkers; 2021; and ASTM E2315-16 Standard Guide for Assessment of Antimicrobial Activity Using a Time Kill Procedure; the time-kill kinetic test was performed with some modifications. The time-kill assay was used against the already mentioned microorganisms at periods of time of 30 and 60 s [8,9,10]. Table 3 describes the challenge of suspension initial population and number control population recovery. Formulations (blank; triclosan 0.03% w/w and xylityl sesquicaprylate 0.45% *w*/*w*) were used without dilution in the presence of 10.0% *v*/*v* fetal bovine serum. The experiment was performed in replicates of three (*n* = 3).

## 4. Conclusions

The xylityl sesquicaprylate-containing alcohol-free mouthwash (0.45% *w*/*w*) presented a particularly interesting profile of efficacy, as a biodegradable antiseptic ingredient, when challenged by the time-kill kinetic test for *A. viscosus*, *F. nucleatum*, *P. gingivalis* and *T. forsythia* (microorganisms that can be involved in caries and periodontal diseases), with results of greater magnitude to reduce the log_10_ of those microorganisms in comparison to the triclosan sample.

## 5. Patents

Xylitol esters and ethers applied as alternative emulsifiers, solvents, co-emulsifiers, and preservative systems for pharmaceutical and cosmetic products; US 8,716,506 B2; 2014.

## Figures and Tables

**Table 1:** Log_10_ reductions of the microorganisms from the time-kill kinetic test for the mouthwash formulations.

Microorganism	Time (s)	Blank-Sample (log10)	Triclosan-Sample (log10)	Xylityl Sesquicaprylate-Sample (log10)
*Actinomyces viscosus*	30	0.03	0.05	***0.20***
	60	0.02	0.07	***0.58***
*Candida albicans*	30	0.00	0.00	0.00
	60	0.00	0.00	0.00
*Fusobacterium nucleatum*	30	0.07	0.04	***1.08***
	60	3.06	2.86	***4.01***
*Klebsiella aerogenes*	30	0.04	0.05	0.05
	60	0.01	0.02	***0.09***
*Porphyromonas gingivalis*	30	1.58	1.82	***4.81***
	60	2.98	2.67	***4.81***
*Prevotella intermedia*	30	1.71	2.98	***4.81***
	60	3.84	***4.41***	3.88
*Streptococcus mutans*	30	***1.48***	1.18	0.41
	60	***2.05***	1.83	0.92
*Tannerella forsythia*	30	2.93	1.83	***5.60***
	60	4.28	4.60	***5.60***

**Table 2:** Active ingredients (% *w*/*w*) of the mouthwash samples (blank, containing triclosan or xylityl sesquicaprilate) *.

Active Ingredients	Composition (% *w/w*)		
	Blank-Sample	Triclosan-Sample	Xylityl Sesquicaprylate-Sample
Triclosan	N.A.	0.03	N.A.
Xylityl sesquicaprylate	N.A.	N.A.	0.45

* Alcohol-free mouthwash qualitative composition: aqua, sodium benzoate, sorbitol, sodium lauryl sulfate, sodium saccharin, sodium fluoride, flavor, PEG 40 hydrogenated castor oil, propylene glycol and CI 42051.

**Table 3:** Challenge suspensions initial population (log_10_) and numbers control population recovery (log_10_) for the microorganisms used in the time-kill kinetic test.

Microorganism	Challenge Suspension Initial Population (log_10_)	Number Control Population Recovery (log_10_)
*Actinomyces viscosus*	10.37	8.41
*Candida albicans*	8.84	6.93
*Fusobacterium nucleatum*	8.31	6.35
*Klebsiella aerogenes*	9.86	7.98
*Porphyromonas gingivalis*	8.67	6.81
*Prevotella intermedia*	8.39	6.41
*Streptococcus mutans*	9.18	7.16
*Tannerella forsythia*	9.79	7.60

## Data Availability

Not applicable.

## Sample Availability

Samples of the compounds are available from some of the authors.
